# Medicine

**Published:** 1903-09

**Authors:** G. Parker


### Progress of the Medical Sciences.

### MEDICINE.

It has been remarked that a new view of the power of self-adaptation by the human organism to its surroundings has been given us by recent investigations in two very different directions. On the one hand, Pawlow and his followers have shown that the digestive organs, and especially the pancreas, elaborate just such ferments as are appropriate to the particular food which stimulates them, and that therefore these vary according to the diet actually taken. On the other, the blood manufactures an infinite variety of lysins and antitoxins when the corresponding toxins are injected into the stream. The latter reaction has not only afforded the means of combatting the infectious fevers, but it has yielded **the biological test for blood**, a means of detecting human and indeed other bloods, as, for instance in stains. The importance of this in forensic medicine is evident at first sight, for it has been thus possible to distinguish human from animal blood which has been shed for thirty years. Even septic processes do not interfere with the reaction.[1] Many workers in this country and abroad have been occupied with the details, and quantitative measures of the reaction have been obtained. The foundation of the whole matter lies in the fact that the serum of an animal which has been several times inoculated with serum from a human being or from a given species of animal will give a "precipitum" when brought into contact with the blood of a human being or of that one species of animal and with no other. Thus serum prepared for human blood reacts only to a drop of human blood whether dried or fresh, that for horses only to equine blood, that for a bird only to the blood of that species.

The reacting bodies in the various bloods and those in the sera of the inoculated animals are remarkably stable, so that both the sera and the dried blood stain may be kept for years unimpaired. Even when the blood is mixed with earth, fatty matters, and various chemicals the reaction can still be obtained; and though a few substances, such as corrosive sublimate or quicklime, prevent the change, it is possible to remove some of these, such as lime, and then to obtain the reaction. Thus the test is available in most conditions where it is needed in legal cases. Again, while a definite precipitum can be obtained in a fixed time with the blood of the animal suited to the serum, Nuttall and Dinkelspiel show that a slight cloud appears with

[1] Graham-Smith & Sanger, *J. Hygiene*, 1903, iii. 258.

the blood of allied species. Thus the "anti-horse" serum reacts only with the blood of the horse, and perhaps the ass, out of tests on the blood of 500 mammals; but a cloudiness is seen on longer exposure with the blood of all mammals, but not of birds, The "anti-lobster" serum reacts with the blood of lobsters, cray fish and five kinds of crabs out of another series of 500 tests, and gives a cloudiness with the blood of more or less allied species, so that the comparative relationships of different groups and orders can be distinguished. Furthermore, the various fluids of the human body can be detected if we have "antisera" which react to each. Martens, finding that a serum produced by injections of albuminous urine reacted to blood, argued that the albumen must be a blood and not a kidney product. However, this deduction needs verification; but the reliability of the test for distinguishing the blood of one animal from another seems to be beyond reasonable doubt.

As a parallel we may turn now to some of the recent work which has been done on the **pancreatic secretions.** It has been shown that each special kind of food leads to the secretion of a digestive juice fitted to act on it. Thus a flesh diet is followed by the secretion of very little trypzymogen, while bread or potatoes produce a large amount.[1] Walther found a fatty diet led to the secretion of a juice rich in steapsin, which breaks up the fat; while Weinland discovered that a milk diet caused the appearance of lactase, which splits up milk-sugar, and is not usually present when other diet is given. Similar selective power is shown in the production of salivary and gastric secretions. The pancreas, however, not only secretes various substances which play an active part in digestion, but it shares in the subsequent metabolism of the carbo-hydrates which have been absorbed. Since Minkowski and others showed that removal of the pancreas was followed by a form of diabetes, some action of the kind has been suspected. Of late it has been argued that this "internal secretion" is due to "certain groups of polygonal cells arranged in columns known as the islands of Langerhans."[2] These are grouped round the blood vessels, and probably represent the primitive pancreas, which has subsequently taken up in addition digestive functions. In certain cases where these islands are affected by what Opie calls interacinar pancreatitis, diabetes results. On the other hand, diabetes does not occur when the whole gland except these islands has degenerated, as in cases of obstruction of the common duct. Thus the islands appear to be the source of a sugar-destroying substance. Lepine had shown that such a substance exists in the blood, and A. C. Croftan[3] has apparently proved that it is contained in the leucocytes, and is probably identical with trypsin. Now, neither this nor the other secre-

[1] *Brit. M. J.*, 1903, ii. 207.
[2] Moynihan, *Practitioner*, 1903, lxxi. 256.
[3] *Am. J. M. Sc.*, 1902, cxxiii. 150; and *Med. News*, 1902, lxxx.

tions of the pancreas can split up sugar or glycogen in pure solutions; but Croftan shows that in the presence of hæmoglobin this ferment does split it up, and at the same time forms bile pigment and bile acids. Thus carbo-hydrate metabolism or glycolysis depends on the liberation of hæmoglobin in the presence of a form of trypsin produced in the pancreas and carried everywhere by the leucocytes. One cause of diabetes then is the failure of the pancreas to supply this ferment for the normal metabolism. It is impossible here to discuss the inhibitory power over this function of the pancreas which is possessed by the suprarenals. Herter[1] and his colleagues have of late worked at this regulatory mechanism, showing that stimulation of the suprarenals, or the application of a minute drop of adrenalin to the bared pancreas is followed by glycosuria. However, the subject is at present an obscure one, and the action of the suprarenals may not be truly inhibitory after all, but counterbalancing only.

**Bovine tuberculosis.**—The great question as to the communication of this disease to man is by no means set at rest, and the direct proof of its conveyance by food presents almost insurmountable difficulties. It is said on the one side that the bacillus of tubercle in men and animals is the same, though modified by the habitat in which it grows, and on the other that there are two distinct organisms, neither of which can be made to grow except in its proper host. Some writers, like Nathan Raw, accept the duality, but contend that tuberculosis in men is due to both organisms. Indeed, the latter author, in his paper at Swansea, takes the extreme view that the majority of cases of tuberculosis in this country are due to bovine infection through milk. He obtained from Koch some cultures of the bovine bacillus, and while he confesses that its morphological characters are very different from those of the human form, he claims that bacilli grown from tuberculous joints and mesenteric glands in the human being show just these pecularities, and that these diseases, and probably lupus, are due to bovine infection, while true phthisis, on the other hand, is the effect of the human variety of the bacilli.[2] Here we have the old distinction of struma and phthisis placed on a bacteriological basis, though it is difficult to accommodate the theory to the numerous facts accumulated in the last thirty years in favour of the identity of the two classes of affections.

One of the very few points on which all parties are agreed is that, if bovine tuberculosis occurs in men at all, it is usually introduced through the alimentary canal.

Thus Koch argues (1) from the absence of great outbreaks

[1] *Med. News*, 1902, lxxx. 865; lxxxi. 769; abstract in *Med. Chron.*, 1902–03, xxxvii. 66.

[2] *Brit. M. J.*, 1903, i. (Jan. 31).

where many persons have drunk tuberculous milk, and (2) from the rarity of primary intestinal lesions.

Can we then separate these alimentary cases from those due to air infection? On this point opinions differ widely. It has been shown that bacilli may pass through the intestinal wall to the mesenteric glands without affecting the former. Hence the first lesion need not be in the intestine.

Ravenel[1] would go further, and thinks that food infection may take place through the tonsils, and again that the bacillus may pass through the intestine, the mesenteric, and other lymph glands to the lung, where the first lesion may occur, and that the fact of the chief enlargement being that of the bronchial glands does not point to the lung as the primary entrance. Price-Jones[2] takes a narrower view. He divides all ordinary cases into Pulmonary, Alimentary, and Generalised. The first have enlarged bronchial glands without obvious disease elsewhere, and he denies that these cases can arise from food infection. The Alimentary type shows enlarged mesenteric glands and none elsewhere, while the Generalised type is of uncertain and indistinguishable origin. The statistics of numerous English and American writers are vitiated for the want of a common definition of cases of Alimentary origin; but taking his own rigid definition, he gets 25 per cent. of the deaths in children as of this type. This, of course, is far above the German, and indeed above most American estimates of its frequency, though other English authorities put it at the same figure and even higher.

The Royal Commission on Tuberculosis is at the present time collecting, through pathologists in various centres, reports of cases due to intestinal infection, so that ere long more definite facts may be looked for.

The question, however, arises, Are these Alimentary cases due to swallowed human bacilli or to bovine infection through milk? Do bacilli isolated from any of them resemble in their mode of growth those of pulmonary origin? or are they like those obtained from cows with tuberculous udders, for even those found in milk may have fallen in it from human sources? The possibility of an answer depends on whether or no bovine bacilli lose their peculiarities after passing through a human being.

Apart from food infection, the transmission of bovine tuberculosis to men by wound infection has been also denied. Ravenel, however, quotes several well-authenticated cases, and Spronck and Koefnagel[3] report an instance where a butcher cut his finger in working on a tuberculous animal. The sore became an ulcer, and produced an enlarged gland at the elbow. From the sore and the gland inoculations were successfully made into guinea pigs, and the disease was then transmitted to others

[1] *J. Tuberculosis*, 1903, v. 69. [2] *Practitioner*, 1903, lxxi. 191.
[3] *Birmingh. M. Rev.*, 1903, liii. 59.

and to a calf. However, an unexceptional proof in such cases is clearly difficult to obtain, for secondary infection of a sore from another source is always possible.

The next point is, Can human tuberculosis be transmitted to cattle? Koch and Baumgarten deny this, but they are testing the matter exhaustively in Germany.[1] They reject as faulty the methods of Arloing and others, and claim that only subcutaneous injections of small amount are trustworthy; neither feeding with tuberculous matter nor injections into the veins or peritoneum are of value. Thus Arloing by the intravenous route infected twenty-three animals with human tubercle.[2] Schottelius infected three oxen by feeding them on human sputa with every precaution against other infection. Fibiger and Henson infected eight cattle by injections, five of which were apparently subcutaneous, and Ravenel in America was equally successful.[3] More recently in Scotland Hamilton and M'Lauchlan Young have succeeded in disproving Koch's assertion while complying with his conditions.[4] Besides other cases infected by food, they transmitted the bacilli to five calves by subcutaneous injections with minute precautions, the animals and the food on which they were fed being previously tested. When the inoculated calves developed tuberculosis, the disease was next transmitted to others by injection, showing that the results were not produced by dead human bacilli and their toxins, but that actual growth had taken place in the calves. Indeed, human bacilli thus ingrafted on a calf gain enormously in virulence when inoculated on a second one. They also find that the morphological characters are no guide to the source of the bacillus, and hold that their results are in favour of the identity of the human and bovine bacilli.—*(Report to the Highland Agricultural Society.)*

On the whole, then, there seems strong reason to believe that bacilli can be transmitted from men to animals, or *vice versâ*, and flourish in their new habitat, but whether such transmission by milk and similar foods is frequent has not yet been decided, and indeed no certain means of solving the question appears to have been found. Even Koch's argument from the absence of epidemics where tuberculous milk has been widely drunk may be met by reference to the millions of persons who fail to contract phthisis, though daily absorbing hosts of bacilli from phthisical patients.

GEORGE PARKER.

### SURGERY.

**Primary Carcinoma of the Vermiform Appendix.**—Dr. A. W. Elting[5] has looked up the original reports of recorded cases of carcinoma of the vermiform appendix, and finds that although

[1] *J. Tuberculosis*, 1903, v. 1, 41. [2] *Bull. de l'Acad. de Méd.*, 1901, xlvi. 897.

[3] *Univ. Penna. M. Bull.*, 1902–03, xv. 66.

[4] Quoted in *Hospital*, 1903, xxxiv. 75. [5] *Ann. Surg.*, 1903, xxxvii. 549.

